# Comparison of Four Search Engines and their efficacy With Emphasis on Literature Research in Addiction (Prevention and Treatment)

**DOI:** 10.5812/ijhrba.6551

**Published:** 2013-03-12

**Authors:** Gholam Reza Samadzadeh, Tahereh Rigi, Ali Reza Ganjali

**Affiliations:** 1 Department of Library and Medical Information Sciences, Zahedan University of Medical Sciences and Health Services, Zahedan, IR Iran; 2 Department of Library and Information Sciences, Zahedan Payame Noor University, Zahedan, IR Iran; 3 Research Center of Children and Adolscents Health, Health Services, Zahedan University of Medical Sciences, Zahedan, IR Iran

**Keywords:** Research, Search Engine, Substance-Related Disorders

## Abstract

**Background:**

Surveying valuable and most recent information from internet, has become vital for researchers and scholars, because every day, thousands and perhaps millions of scientific works are brought out as digital resources which represented by internet and researchers can’t ignore this great resource to find related documents for their literature search, which may not be found in any library. With regard to variety of documents presented on the internet, search engines are one of the most effective search tools for finding information.

**Objectives:**

The aim of this study is to evaluate the three criteria, recall, preciseness and importance of the four search engines which are PubMed, Science Direct, Google Scholar and federated search of Iranian National Medical Digital Library in addiction (prevention and treatment) to select the most effective search engine for offering the best literature research.

**Materials and Methods:**

This research was a cross-sectional study by which four popular search engines in medical sciences were evaluated. To select keywords, medical subject heading (Mesh) was used. We entered given keywords in the search engines and after searching, 10 first entries were evaluated. Direct observation was used as a mean for data collection and they were analyzed by descriptive statistics (number, percent number and mean) and inferential statistics, One way analysis of variance (ANOVA) and post hoc Tukey in Spss. 15 statistical software. P Value < 0.05 was considered statistically significant.

**Results:**

Results have shown that the search engines had different operations with regard to the evaluated criteria. Since P Value was 0.004 < 0.05 for preciseness and was 0.002 < 0.05 for importance, it shows significant difference among search engines. PubMed, Science Direct and Google Scholar were the best in recall, preciseness and importance respectively.

**Conclusions:**

As literature research is one of the most important stages of research, it's better for researchers, especially Substance-Related Disorders scholars to use different search engines with the best recall, preciseness and importance in that subject field to reach desirable results while searching and they don’t depend on just one search engine.

## 1. Background

Surveying the valuable and the most recent information has become vital for researchers and scholars, because every day, thousands and perhaps millions scientific works are bought out as digital resources that represented by internet and researchers can’t ignore this great resource for literature review and they find related documents for their literature searche that may not be found in any library (1). Developing new technologies, scholars have faced abundant variety of collection literature research review with regard to subject of presentation and information space. Web cyberspace attracts the researchers’ attention because of variety of data with different forms and simultaneity. Literature search review is one of the most important search stages. Revising the literature search detected by search station in the broader tissue off helps researcher to expand the research view and his/her landscape. On the other hand he/she limits the research title and reaches to an intensive research question (2). Web, a huge resource of data plays an important role as an information reference for scholars. Nowadays, the rate of network data growth caused inaccessibility of information that is more worse than lacking them (3). With the creation and development of internet network as the greatest, the most varied and the most widespread information resource, simultaneous and various types of search tools are appeared to help users find information they want, these tools include search engines, meta-search engines and subject directories (4). Information retrieval is a challenge for users since search tools are too complex to navigate (5). As one of the most effective searching tools, its role is distinct because those search engines use software facilities which can find information from different sites for users. On the other hand, during the past decades developed knowledge in Substance-Related Disorders caught everyone’s eyes enormously (6). This growth is as a result of discussing about addiction as a national, regional and multi-dimensional problem. The volume of produced information and up to date personal data necessitates to study hours in a day, even a small part of this subject. Therefore, persuading to use internet is essential for updating data literature review. Then we concluded that the mentioned four popular search engines in medical sciences include: PubMed, Science Direct, Google Scholar and federated search of Iran’s health, treatment and medical education ministry and indicated that whether the of above mention search engines offered the best literature search about addiction (prevention and treatment). In Tober’s study, the four most popular search engines; PubMed, Science Direct, Scopus and Google Scholar are investigated to evaluate which search engine is the most effective for literature research in laser medicine. He concludes that all in all, Scopus was the most effective search engine in the literature research ,in case of requiring only an overview of the topic, even for a widespread and in depth investigation in area of life sciences and closely related topics, PubMed was more appropriate. Google Scholar, Science Direct are the best in preciseness and importance criterion in laser medicine (7). Also Bajpaie and et investigated in a research and compared 18 search tools, their results showed that four tools could be better than the others; these tools include two of the full-text scanners (High wire press & Google Scholar) and two citation scanners (PubMed & Scopus). The results show that, use of a single search tool can lead to loss of up to 70 % of the relevant citations in some cases. Hence, use of multiple search tools is recommended (8). Anders & Ivans compared PubMed with Google Scholar literature search in respiratory care topics by cross-sectional study and their research results showed that PubMed and Google Scholar had similar recall, but at precision criterion PubMed was better than the other. According to researches, PubMed was more efficient and better than Google Scholar with regard to the patient's searches and educational purposes (9).

In research by Flagas et al. a Comparison was made to evaluate the strength and weaknesses of PubMed, Scopus, Web of Science, and Google Scholar. Results showed that all databases were practical and offered numerous search facilities. PubMed and Google Scholar were free access for users. PubMed had offered optimal update frequency and include recent online articles; other database had rated articles based on some criteria such importance. For citation analysis, Scopus offered about 20% more coverage than Web of science; and Scopus covers the wider range of journals. Google Scholar can help in the retrieval of even the most oblique information and less often update (10). In a descriptive research, Mohammad Esmaeil, Lafzghazi and Gilvari compared six search engines and six meta-search engines in pharmaceutics information retrieval, results showed that if users survey in several search engines, they access to the relevant documents among the vastly available sources on web. Their research showed that Yahoo retrieved the most pharmaceutics documents and AOL had 62% precision and 21% recall, it retrieved the most relevant pharmaceutics documents. Among meta-search engines Dogpile was better than others (4). Outline description from evaluated search engines is explained in in the following:

### 1.1. PubMed

In 1997, PubMed (http://www.ncbi.nlm.nih.gov/PubMed) was offered by the National Library of Medicine on internet. It is one of the most popular and the most responsible resources on the World Wide Web for physicians and scholars (10). PubMed is a free search engine to search about medicine and biomedical journal literature. It searches several databases and interfaces Medline, directly. This search engine maps user’s search terms to the Medical subject heading (Mesh) and text words in Medline records and then searching (9). The PubMed offers users numerous powerful searche filters to limit their searches and gives them desirable retrieval information (11).

### 1.2. Science Direct

Science Direct (http://www.science direct.com) is a full text scientific database which is a part of the science verse and is provided by Elsevier publication in 1997. The web portal of Science Direct opens with features which invites the users just to browse the word scientific publications (7). This search engine is one of the greatest bibliographic and full text electronic collections about science, technology and medicine. Also we can have an exact searching with regard to limitations and abilities that is offered by Science Direct (12).

### 1.3. Google Scholar

Google Scholar (http://scholar.google.com): it’s design and handling is similar to that of Google search engine. It provides a simple way to search broadly for scholarly literature. In a particular place you can search across many disciplines and sources such as articles, theses, books, abstract and etc. This search engine helps you to find relevant scientific works in all over the world of sceince (12). The search results in Google Scholar can be limited to title, author, publication source, publishing date and other filters (13).

### 1.4. Integrated Digital Library (IDL):

IDL is an advanced system which gives us simple and one step access to all electronic resources at the Iranian National Medical Digital Library. Also we can browse and search all databases, e-journals, e-books and references in digital library alphabetically or by subject. Federated search allows you to search multiple online databases. Federated search saves your time and provides your favorite results quickly (14).

## 2. Objectives

The aim of this study is to evaluate the three criteria such as recall, precision and importance in the four search engines: PubMed, Science Direct, Google Scholar and federated search of Iranian National Medical Digital Library in addiction (prevention and treatment) to select the most effective search engine for offering the best literature research.

## 3. Materials and Methods

This research was cross-sectional study and we evaluated four famous and popular search engines in medical sciences include: PubMed, Science Direct, Google Scholar and federated search in IDL. We limited search to “Substance-related disorders” keyword in all search engines because this was the most common key word that specialists used. Documents that search engines offer are listed as results according to relevant value of query search with descent sequence. Hence, document offered as the first record in the search results, is the most related document to query search, from vision of that search engine (3). With regard to what was mentioned above and what cited to Tober’s study, sample research selected 10 first results of retrieval documents of each search engines. To compare search engines, three criteria, recall, precision and importance were evaluated (7). The calculation method for each criterion is explained as follow:

### 3.1. Performance test: Criteria of evaluation

The aim of the performance test was to get an overview about article dealing with the addiction topic (prevention and treatment). Therefore, we consulted with psychiatrist and selected proper key word among prevalent terms, and then matched with web version of mesh (15), and “Substance-Related Disorders” keyword was chosen as the most relevant query search at search engines in addiction (prevention and treatment).

1. The criterion ‘‘recall’’ (or ‘‘hits’’) is the number of found articles and is related to the integrity of the evidence base.

2. The criterion ‘‘precision’’ determines how well the filtered articles cover the topic of the search term and influences the time and cost of screening and the results for the related articles. It is evaluated by counting the search term in the search fields ‘‘title’’ (n title) and ‘‘abstract’’ (n abstract). The appearance of the terms in the field ‘‘title’’ (a) is two times more than the field ‘‘abstract’’ (b). Additionally the appearance is rated by the rank i, the position of the filtered article in the results list. From this the precision P can be calculated as follows:

P = ( (n + 1) – i ) • (a × n title + b × n abstract)

3. The criterion ‘‘importance’’ is determined by the number of citations “n” citation by publications of other authors. For this articles, citations that were in Science Direct and Google Scholar had been considered and for articles, citations were offered by PubMed and IDL and the other articles without citation, Web of knowledge citation database was used. Number of citation was used for calculating importance criterion and the rank i as described before. From this, the importance I is calculated as follows:

I = ((n + 1) − i) • n citation

For this study, only English written abstract and title which contain the search term “Substance-Related Disorders” were analyzed, for collecting information and data, directly observation was used. We entered keyword in each search engine, and to evaluate criteria, number of all retrieval articles (hits), titles, abstracts, and citations of 10 first articles in the result list were considered. All searches were conducted on November 29th, 2011 on Windows seven environment with Microsoft Internet Explorer in the Central Library of Zahedan Medical Sciences University. We used statistical software Spss 15 for data analysis. One-way analysis of variance (ANOVA) and post hoc Tukey was performed to evaluate search engines. P < 0.05 was considered statistically significant.

## 4. Results

Results showed that PubMed retrieved most of the documents, 213 articles (32%) and it was better than the other search engines on recall criterion. Results from recall criterion were similar in Science Direct with 188 hits (28%) and, Google Scholar with 184 hits (27%). And IDL with 91 hits (13%) offered the least articles in this subject (Table 1).

**Table 1:** Number of Hits or Recall

Recall (hits)	Search engines
**PubMed**	213
**Science direct**	188
**Google scholar**	184
**IDL**	91

After survey of titles and abstract precision and statistical indicators of each search engines were investigated. Investigation of means showed that “the most precision” has allocated to Science Direct search engine with mean 25.2 (33%) and after that was ILD with mean 17.9 (24%), and then were PubMed with mean 16.2 (22%) and Google Scholar with mean 16 (21%). For detecting precision among search engines, One-way analysis of variance (ANOVA) was used and P value earned (0.726) which was more than 0.05, and this explained there was not statistically significant difference among search engines for precision. Whereas assessed P value from article titles investigation was P = 0.004 which showed significant difference in precision among search engines. Post hoc Tukey showed that there was difference between Science Direct with PubMed (Table 2).

**Table 2:** Results for the “Precision”

Ranking	IDL			Google Scholar			Science Direct			PubMed		
	P	Abstract, No.	Title, No.	P	Abstract, No.	Title, No.	P	Abstract, No.	Title, No.	P	Abstract, No.	Title, No.
**1**	10	1	0	50	3	1	60	4	1	20	2	0
**2**	18	2	0	18	0	1	72	6	1	81	7	1
**3**	64	5	1	32	2	1	32	2	1	8	1	0
**4**	7	1	0	0	0	0	28	2	1	14	2	0
**5**	6	1	0	18	1	1	24	2	1	6	2	0
**6**	35	3	2	15	1	1	10	1	1	20	2	1
**7**	8	2	0	8	0	1	8	1	1	4	1	0
**8**	12	2	1	9	1	1	6	1	1	3	1	0
**9**	18	7	1	6	1	1	8	2	1	2	1	0
**10**	1	1	0	4	2	1	4	2	1	4	2	1
**Mean**	17.9			16.0			25.2			16.2		

For importance, results showed that Google Scholar was the best and its means was 510.3 (82%), and after that was Science Direct, with mean 115.6 (18%). IDL with mean 0.3 (0%) and PubMed were not scored. Results of One-way analysis of variance (ANOVA) showed P Value = 0.002 that was less than 0.05, so we used Post hoc Tukey. Finding from importance criterion showed statistically significant difference between Google Scholar with each of the others search engines (Table 3). 

**Table 3:** Results for the “Importance”

Ranking	IDL		Google Scholar		Science Direct		PubMed	
	I	Citation, No.	I	Citation, No.	I	Citation, No.	I	Citation, No.
**1**	0	0	1350	135	1050	105	0	0
**2**	0	0	1125	125	0	0	0	0
**3**	0	0	512	64	56	7	0	0
**4**	0	0	1211	173	14	2	0	0
**5**	0	0	438	73	18	3	0	0
**6**	0	0	205	41	0	0	0	0
**7**	0	0	128	32	0	0	0	0
**8**	3	1	81	27	0	0	0	0
**9**	0	0	36	18	8	4	0	0
**10**	0	0	17	17	10	104	0	0
**Mean**	0.3		510.3		115.6		0.0	

## 5. Discussion

Literature search is one of the most important steps in the research process. We review literature to avoid heavy works, to ensure we have a thorough understanding of the topic, to identify similar work done within the area, to identify knowledge gaps that demand further investigation, to compare previous findings, to critique existing finding and suggest further studies (16), which this features increase the importance or the exact literature review. Review of literature accomplishes for literature search in medical sciences on the internet by different search engines that they were compared, evaluated and reviewed by the other researchers in different countries to identify their search’s features and abilities (5-10) (15-22). This research was done to select the most effective search engine for offering the best literature research in addiction (prevention and treatment).

Investigations showed that search engines in various subject fields with regard to unique abilities and facilities are done differently, which this affair supported by other researchers, too (16, 18). Search engines cover just a limited part of accessible information on the web and neither of them don’t have total recall and precision. The scale of recall, precision (23), and importance of search engines are different with regard to purpose of researchers from search. Results of this study showed that evaluated criteria were different in search engines, whereas PubMed had the most recall criterion and then were Science Direct and Google Scholar with similar recall. Recall in PubMed was about two times more than ILD’s recall. The results of Tober study were different, whereas PubMed had least recall in laser medicine (7). At the other research was done in respiratory care, results showed that the function of two search engines (PubMed & Google Scholar) was similar in recall criterion (9), our study showed this similarity between Google Scholar and Science Direct.

Means investigation explained that Science Direct offered the best precision and related documents in addiction. Although Results of One - way analysis of variance (ANOVA) did not show significant difference in precision among search engines, results were different in investigation of retrieved articles titles which P - value was less than 0.05 that explanatory existence showed significant difference among search engines, post hoc Tukey showed that considerable difference exists between Science Direct and PubMed. Also, in Tober study, Science Direct after Scopus had the best precision 7. But in the other research, results were different whereas PubMed had above precession (9).

In this study, Google Scholar had maximum importance and the next was Science Direct. PubMed and IDL were not scored. In PubMed Tober’s study, results were similar to our study in this criterion. For importance, results of ANOVA showed significant difference, moreover post hoc Tukey and comparing means showed that the difference was remarkable among Google Scholar. Also, former researcher supported indication of Google Scholar for offering the citations which caused importance of articles (10, 20, 22). Reviews of this research showed that IDL did not offer acceptable results in any of three criteria, with regard to expensive cost that Iran’s health, treatment and medical education ministry pay for funding medical sciences universities and this will be revision to buy and use this database or be changed the operation of federated search.

We had local limitation for our searches in federated search of IDL and Science direct because this search engines were not available to the public. The other limitation to our study was that we only used the “Substance- related disorders” keyword in search engines and didn’t use the related keywords. Next limitation was that at first, we wanted to evaluate Scopus along with the other search engines, but Scopus was disconnecting in Iran and thus excluded from this study. Results showed that use of one search engine for literature review in addiction (prevention and treatment), neither proper nor search engines can’t give us the best results. Former studies, also recommended combined search and simultaneous use from several search engines to reach effective and related results (4, 8, 17). With regard to broad extent, multilateral addiction subject and new sights discussion, it is recommended that researchers must use different search engines with the best recall, precision and importance to reach the best results, they must continue their search with abilities and various services that search engines give.
